# In Reply

**DOI:** 10.1093/oncolo/oyaf227

**Published:** 2025-08-13

**Authors:** Frédérique Penault-Llorca, Mark A Socinski

**Affiliations:** Department of Pathology, Centre Jean Perrin, Université Clermont Auvergne, INSERM, U1240 Imagerie Moléculaire et Stratégies Théranostiques, Clermont Ferrand F-63000, France; Oncology and Hematology, AdventHealth Cancer Institute, Orlando, FL 32804, United States

We thank Drs. Nishimura and Fujimoto for their letter entitled “Is molecular testing necessary for squamous cell carcinoma,” as they raise several important considerations.^1^ Precise subclassification of non-small cell lung cancer (NSCLC) is important as the molecular findings in adenocarcinoma vs squamous carcinoma differ. Proper classification has been shown to be linked to the quality of the biopsy specimen, diagnostic confidence, years of experience, and expertise in pulmonary pathology.^2^^,^^3^ When there is a lack of complete clarity of the histologic subtype, we would argue to test, as the identification of an oncogenic driver with approved targeted therapies could significantly change the clinical course of the patient.

Traditionally, the role of molecular testing in squamous carcinoma has been de-emphasized with exceptions based on clinical features such as smoking status. It has been shown that the molecular profile in squamous carcinoma does differ based on smoking status.^4^ However, as therapeutic options for molecularly defined subsets of NSCLC have increased, the importance of testing all patients with NSCLC becomes more relevant. The corollary to this statement assumes that the use of targeted therapies in these molecularly defined subsets has been established. Data on squamous carcinoma are limited, and the existing information is not very promising, although some responses have been documented.^4–7^ We agree with Drs. Nishimura and Fujimoto with regard for the need for more prospective testing of targeted therapies, ideally in the context of clinical trials.

## Disclosures

F.P.-L. reports speaker/consultancy honoraria from AstraZeneca, Daiichi Sankyo, Eisai, Pfizer, MSD, Novartis, Roche, Seagen, Medscape, Gilead, Janssen, Lilly, Astellas, and Amgen; and travel grants from AstraZeneca, Roche, Pfizer, Daiichi Sankyo, Gilead, MSD, and Novartis. M.A.S. reports speaker/consultancy honoraria from AbbVie, AstraZeneca, Bristol-Myers Squibb, Foundation Medicine, Genentech, Gilead, Guardant, Janssen, Jazz Pharmaceuticals, Mirati Therapeutics, Novartis, OncoC4, Regeneron, and Summit. He also reports research funding (institutional) from Cullinan Oncology, Daiichi Sankyo, Genentech, OncoC4, Pfizer, and Spectrum Pharmaceuticals.
